# Relationship between Coronary Artery Calcium Score and Coronary Stenosis

**DOI:** 10.1155/2023/5538111

**Published:** 2023-12-15

**Authors:** Xinyan Chen, Jianbin Zhao, Qingqing Cai, Rong Chen, Wenhao Wu, Peng Wang, Gaoxing Zhang, Jinhuan Zhen

**Affiliations:** ^1^Department of Cardiology, Guangdong Medical University, Zhanjiang, Guangdong 524000, China; ^2^Department of Cardiology, Jiangmen Central Hospital, Jiangmen, Guangdong 529000, China; ^3^Department of Network Information, Jiangmen Central Hospital, Jiangmen, Guangdong 529000, China; ^4^Department of Cardiology, Kaiping Second People's Hospital, Jiangmen, Guangdong 529300, China

## Abstract

**Background:**

The coronary artery calcium score (CACS) is commonly employed to quantify the degree of calcification in coronary atherosclerosis. Indeed, increased coronary stenosis severity is associated with a progressive increase in CACS.

**Objectives:**

This study sought to explore the association between CACS and coronary stenosis of ≥50% and ≥70%.

**Methods:**

We conducted a retrospective analysis of patient data collected between July 1, 2017, and March 3, 2022, at Jiangmen Central Hospital. A total of 208 patients, presenting with both symptomatic and asymptomatic manifestations and suspected coronary artery disease (CAD), were included. Statistical analyses included ROC curve assessments, subgroup analyses based on age, and comparisons of CACS values against the presence of coronary stenosis ≥50% and ≥70%.

**Results:**

Ultimately, 208 patients were included, with a median age of 65.0 years and a median CACS of 115.7 (interquartile range: 13.7–369.4). A CACS threshold of ≥1300 demonstrated a specificity of 100% for coronary stenosis of ≥50%. Notably, the percentage of patients with obstructive CAD showing CACS = 0 was significantly higher in those under 65 years (15.1%) compared to patients over 65 years (3.8%) (*P*=0.005). The inflection point, at which the risk probability for coronary stenosis of ≥50% shifted from being a protective factor to a risk factor, was observed when CACS fell within the range of 63.3 to 66.0.

**Conclusion:**

CACS demonstrates good performance for the detection of coronary artery stenosis.

## 1. Introduction

It is well established that atherosclerotic cardiovascular disease (ASCVD) exhibits a high prevalence and mortality rate globally. In China, a total of 2.4 million deaths were attributed to ASCVD alone in 2016, accounting for approximately 25 percent of all mortality cases. Coronary calcification is a component of atherosclerosis [1]. For asymptomatic patients, if a patient presents with multiple risk factors, such age, family history, smoking, diabetes, hypertension, and abnormal lipid levels, healthcare providers may consider CCTA as a noninvasive imaging modality to assess the health of the coronary arteries [2]. The past few years have witnessed the advent of the coronary artery calcium score (CACS) to quantify coronary calcification and predict the presence of coronary artery stenosis [3]. Indeed, the severity of stenosis is increased with an increase in CACS [4]. The prognostic outcome can be predicted by CACS with a deterioration in prognosis corresponding to an increase in CACS [5]. It has been reported that the 10-year event rates of CACS values of 0, >100, and >300 were 5.6%, 7.5%, and 13.1%, respectively.

Current guidelines for coronary artery revascularization define significant stenosis as non-left main disease diameter stenosis ≥70% and left main stenosis ≥50% [6]. Palumbo et al. suggested the use of CACS as a preliminary screening tool for coronary computed tomography angiography (CCTA) [7]. Alshumrani employed various cutoff values of CACS to assess coronary stenosis ≥50% and ≥70%, revealing that all symptomatic patients had coronary stenosis ≥50% when CACS was ≥250 [4]. However, it is worth noting that this previous study did not employ coronary angiography (CAG) as a criterion for evaluating the severity of coronary stenosis. It should be acknowledged that high cutoff values for CACS may lead to an overestimation of the extent of coronary stenosis [8]. The present study employed CAG for diagnosing coronary stenosis, encompassing both symptomatic and asymptomatic patients with suspected CAD. The objectives of this study were as follows: (1) To assess the diagnostic value of CACS for coronary artery stenosis ≥50% and 70%. (2) To investigate the impact of age on CACS-based diagnosis of coronary stenosis. (3) To analyze the relationship between CACS and the risk probability of coronary stenosis ≥50% and 70% using restricted cubic spline (RCS).

### 1.1. Patient Data

This study was approved by the Ethics Committee of Jiangmen Central Hospital ([2022]56). Informed consent was not required, given that anonymous patient data was used in the study. This retrospective study focused on patients who underwent CCTA and subsequent CAG at Jiangmen Central Hospital between July 1, 2017, and March 3, 2022. The study population consisted of patients presenting with either symptomatic or asymptomatic manifestations but exhibiting suspected CAD. The observed symptoms encompassed angina, dyspnea, syncope, or palpitation. Exclusion criteria are as follows: Patients who had undergone percutaneous coronary intervention (PCI) and coronary artery bypass graft surgery prior to CCTA or lacked CACS data were excluded from the study. Baseline clinical data were collected, including demographic characteristics, nutritional status, past medical and surgical history, laboratory tests, CACS, and CAG results. Comorbidities such as diabetes mellitus and hypertension were defined using the International Classification of Diseases, 10th revision diagnostic codes. All CCTA were assessed by an experienced radiologist who was blinded to the results of CAG. CAG and severity of coronary stenosis were performed and evaluated by an experienced cardiologist who was blinded to the CCTA results.

### 1.2. Computed Tomography

The Siemens Somatom Dual Source Force Computed Tomography was employed for the examinations. The procedure began with a localization phase acquisition, which was followed by a scan to determine the coronary artery calcification score. To enhance imaging quality, nonionic contrast media was intravenously administered via the cubital vein prior to the CT scanning. The scan covered the area from the tracheal bifurcation down to 2 cm below the left diaphragm, with a slice thickness of 0.625 mm. One of the advantages of Siemens Somatom Dual Source Force CT is that it circumvents the need for prescan heart rate control with beta-blockers, producing high-quality images, even in patients with elevated or irregular heart rates. The raw data from the scans are transferred to imaging workstations, allowing for postprocessing techniques such as multiplanar reconstruction, maximum density projection, and 3D volume reproduction. The CACS was determined using Agatston's algorithm.

### 1.3. Coronary Angiography

The cardiologist, who was blinded to the CCTA results, performed a right radial artery puncture using Seldinger's technique and inserted a 6Fr sheath. Heparin (3000 *μ*) was administered prior to conducting left and right coronary angiography with a 6Fr Heartrail angiography catheter. Subsequently, the severity of stenosis in the left main coronary artery (LM), left anterior descending artery (LAD), left circumflex artery (LCX), and right coronary artery (RCA) was evaluated.

### 1.4. Statistical Analysis

The data analyses were performed using the R software. The study population characteristics were presented as mean ± SD (standard deviation) for continuous variables, while categorical variables were expressed in percentages. Continuous variables that were not normally distributed were expressed using the median and interquartile range. Differences in categorical variables were assessed using a Chi-square test. The Pearson test was employed to assess the correlation between continuous variables following a normal distribution, while Spearman's correlation was utilized for examining the correlation among continuous variables with non-normal distribution characteristics. A receiver operating characteristic curves (ROC) curve analysis was performed, including calculations of specificity, sensitivity, positive predictive value (PPV), negative predictive value (NPV), and area under the ROC curve (AUC) for CACS predicting coronary stenosis and was used to identify the best cutoff point by the Youden index. A generalized additive model was used to analyze the relationship between CACS and coronary stenosis. RCS was employed to investigate the functional form of this association. The significance level was set at a two-sided *P* value threshold of <0.05. The difference in the area under the curve was evaluated and tested using the test method proposed by Hanley and McNeil. The severity of coronary stenosis is evaluated using CAG as a reference standard.

## 2. Results

### 2.1. Patient Characteristics

The present study included a total of 208 patients exhibiting male predominance (*n* = 138, 66.3%) who presented symptomatically or asymptomatically with suspected CAD. The age of the entire patient cohort ranged from 33 to 89 years, with a median age of 65.0 years (interquartile range: 58–71 years). The median time interval between CCTA and CAG was 6 days (non-normal distribution, interquartile range 3–20 days). The median duration between CCTA and serum calcium measurements was 4 days (non-normal distribution, interquartile range 2–7 days). The median age of patients with coronary stenosis ≥50% and ≥70% was 66.0 (60.3–70.0) and 65.0 (57.8–71.0) years, respectively, with no significant difference between the two groups (*P*=0.87). Among the cohort of 208 patients, the median CACS was 115.7, ranging from 0 to 2243.6, and the interquartile range was 13.7–369.4 (Table 1).

Coronary stenosis was absent in 16.3% (*n* = 34) of patients. The prevalence of coronary stenosis ≥50% was 79.9% (*n* = 166 patients), and ≥70% was 60.6% (*n* = 114 patients). A total of 829 coronary artery segments were evaluated across all patients (Table 1).

### 2.2. CACS and Coronary Stenosis Rate

Among patients with CACS = 0 (*n* = 32), the mean maximum luminal stenosis was 44.3% (range 0–100%). Including patients who exhibited no evidence of coronary stenosis (*n* = 11, 34.4%), coronary stenosis ≥50% (*n* = 16, 50.0%), and coronary stenosis ≥70% (*n* = 10, 31.3%).

**Figure 1:**
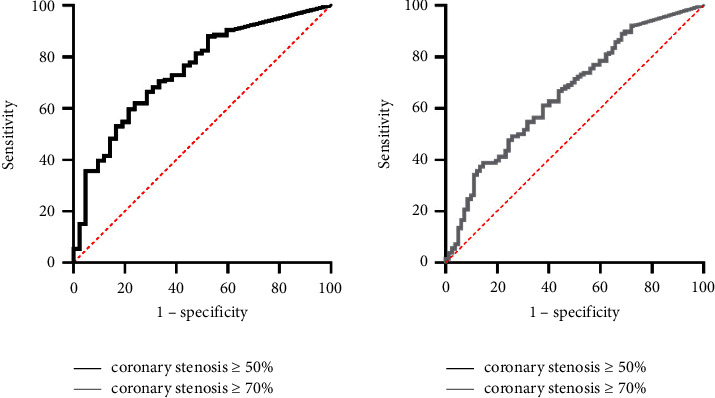
ROC curves for CACS diagnosis of coronary stenosis ≥50% and ≥70%.

According to the ROC curve analysis, the optimal cutoff point of CACS for identifying coronary stenosis ≥50% was 87.0, yielding a sensitivity of 62.0%, specificity of 76.2%, PPV of 91.2%, NPV of 33.7%, and accuracy of 64.9% (AUC = 0.75). The optimal cutoff point of CACS for identifying coronary stenosis ≥70% was 290.8, yielding a sensitivity of 38.9%, specificity of 85.4%, PPV of 80.3%, NPV of 47.6%, and accuracy of 57.2% (AUC = 0.66) (Figure 1).

The diagnostic performance of different cutoff values of CACS was assessed for the detection of coronary stenosis ≥50% and ≥70% (Table 2). Regarding coronary stenosis ≥50%, the CACS cutoff of ≥1300 exhibited a sensitivity of 5.4%, specificity of 100%, PPV of 100%, NPV of 21.1%, and diagnostic accuracy of 24.5%.

### 2.3. Comparison of Diagnostic Performance of CACS in Patients Aged ≤65 years and >65 Years

Patients ≤65 years of age and patients >65 years of age with CACS = 0 but coronary stenosis ≥50% were 15.1% and 3.8%, respectively (15.1% vs. 3.8%, *P*=0.005). The AUC of CACS in predicting coronary stenosis ≥50% did not demonstrate statistical significance among patients ≤65 years and >65 years (0.79 vs. 0.70, *P*=0.22), as well as for coronary stenosis ≥70% (0.67 vs. 0.67, *P* > 0.05).

### 2.4. Association between CACS and the Risk Probability of Coronary Stenosis

**Figure 2:**
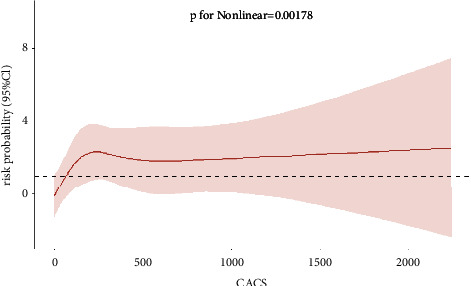
Multivariable-adjusted cubic spline model for the association between CACS and risk probability for coronary stenosis ≥50%.

**Figure 3:**
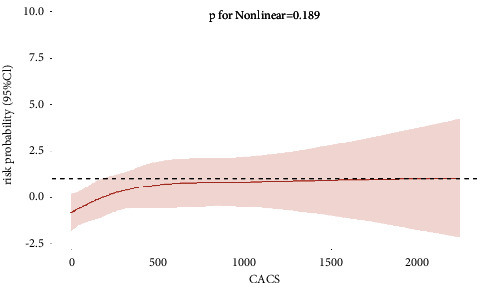
Multivariable-adjusted cubic spline model for the association between CACS and risk probability for coronary stenosis ≥70%.

After adjusting for body mass index (BMI), hemoglobin (Hb), N-terminal pro-B-type natriuretic peptide (NTproBNP), estimated glomerular filtration rate (eGFR), high-density lipoprotein cholesterol (HDL), low-density lipoprotein cholesterol (LDL), triglyceride (TG), cardiac troponin-I (TnI), ejection fraction (EF), systolic blood pressure, diastolic blood pressure, heart rate, total cholesterol, calcium, history of smoking, diabetes mellitus, and hypertension, RCS was used to analyze the association between CACS and the risk probability of coronary stenosis ≥50% and 70%. After adjusting for multiple variables, the cubic spline model showed nonlinear associations between CACS and the risk probability of coronary stenosis ≥50% (*P* for nonlinear associations = 0.002) (Figure 2). CACS and the risk probability of coronary stenosis ≥70% exhibited a linear correlation (total *P* = 0.0172, *P* for nonlinear associations = 0.189) (Figure 3). The inflection point in the risk probability of coronary stenosis of ≥50% was observed within a CACS range of 63.3–66.0 (Figure 2). Beyond this range, a substantial escalation in risk was observed, peaking at 230.8; thereafter, the risk stabilized. In subgroup analyses stratified by age (≤65 years or >65 years), no significant association between CACS and the occurrence of coronary stenosis was found (coronary stenosis ≥50%, *P* = 0.975; coronary stenosis ≥70%, *P* = 0.618).

## 3. Discussion

This study evaluated multiple CACS cutoff values for diagnosing coronary stenosis and investigated the association between CACS and the presence of coronary stenosis. CAG was employed as the reference standard to evaluate the severity of coronary stenosis. Our results revealed that high CACS cutoff values provided a high level of specificity and PPV when diagnosing both ≥50% and ≥70% coronary stenosis. Significantly, CACS = 0 did not consistently exclude the possibility of coronary stenosis of ≥50% or ≥70%, particularly in younger patients. The point of transition in the risk probability of coronary stenosis of ≥50% shifted from being a protective factor to a risk factor when CACS fell within the range of 63.3 to 66.0. In subgroup analyses stratified by age, no significant association between CACS and the occurrence of coronary stenosis was found.

High CACS cutoff values demonstrated high specificity and PPV for the diagnosis of coronary stenosis ≥50% and ≥70%. In this respect, de Agustin et al. showed that the specificity and PPV for detecting coronary stenosis ≥70% in patients presenting with chest pain and CACS ≥400 were 93.5% and 85.8%, respectively [9]. Accordingly, it is recommended that patients experiencing chest pain and having CACS of ≥400 should refrain from undergoing CCTA. Consistently, our study demonstrated a high PPV of 95.7% for detecting coronary stenosis ≥50% when CACS ≥400. Besides, CACS ≥1300 yielded good specificity and a positive predictive value of 100% for diagnosing coronary stenosis ≥50%. Collectively, these findings indicate all patients with CACS ≥1300 had coronary stenosis ≥50%. Such patients can make an informed decision about foregoing CCTA and instead choose early percutaneous coronary revascularization based on their preferences regarding invasive procedures.

Kiani et al. showed that age exerts an impact on the optimal cutoff value [10, 11]. Durhan et al. conducted a ROC curve analysis to assess the predictive ability of CACS for coronary artery stenosis across different age groups, revealing that patients aged 50–59 exhibited a significantly higher AUC than other age groups [12]. However, the above studies failed to consider whether the differences were statistically significant. In the present study, the diagnostic value of CACS in coronary stenosis in the two age groups was assessed by ROC curve analysis. However, the difference in performance of CACS in predicting coronary stenosis ≥50% in patients aged ≤65 years and >65 years was not statistically significant. During subgroup analyses based on age, there was no significant association between CACS and the occurrence of coronary stenosis.

A CACS = 0 does not definitively rule out the presence of coronary stenosis of ≥50% or ≥70%, especially in younger patients. Previous studies have shown that among patients with CACS = 0, a range of 1.5% to 8.3% displayed coronary stenosis of ≥50%, and 1.4% presented with coronary stenosis of ≥70% [13–17]. Feuchtner et al. found that 12.5% of young adults aged 35–49 years with CACS = 0 had coronary stenosis ≥50% [18]. In our study, among patients with CACS = 0, 50.0% exhibited coronary stenosis of ≥50%, while 31.3% had coronary stenosis of ≥70%. Therefore, it can be concluded that coronary stenosis of ≥50% or ≥70% cannot be completely excluded when CACS = 0 in patients with symptomatic or asymptomatic CAD. There are several possible explanations for this phenomenon. First of all, CCTA categorizes plaques based on the presence or absence of calcified components, distinguishing between calcified, mixed calcified, and noncalcified plaques. However, CACS cannot visualize coronary stenosis caused by noncalcified plaque. In patients with CACS = 0, most plaques were predominantly noncalcified [19]. Kelly et al. found noncalcified plaque in 10 of 12 patients with a CACS of zero and coronary stenosis of ≥50% [20]. Besides, it should be borne in mind that the early stages of coronary atherosclerosis do not exhibit any calcification. These results suggest that CCTA or CAG should still be considered to identify coronary stenosis of ≥50% in young patients. Interestingly, Konieczyńska et al. found that women with a CACS of zero demonstrated an NPV of 100% for the presence of coronary stenosis, suggesting that coronary stenosis can be ruled out in symptomatic female patients when their CACS is zero [11, 21]. However, in our study, the influence of gender on the exclusion of coronary stenosis of ≥70% with a CACS of zero remained uncertain due to the male predominance of our study population. Additional research with a larger sample size is warranted to clarify this aspect.

Age is an influential factor in excluding coronary stenosis ≥50% when the CACS is zero [22, 23]. A higher proportion of patients aged ≤65 years with both coronary stenosis ≥50% and CACS = 0 was observed compared to those aged >65 years. The findings of this study align with those reported by Mortensen et al., which showed that among the five age groups of patients with coronary stenosis ≥50%, the proportions of patients with a CACS of zero were 58% for individuals under 40 years, 34% for those aged 40 to 49 years, 18% for those aged 50 to 59 years, 9% for those aged 60 to 69 years, and finally, only 5% for individuals over the age of 70 years, consistent with Albuquerque et al.'s study [22, 23]. The exclusion of coronary stenosis ≥50% using CACS = 0 should be approached with caution in young patients, as its value decreases with decreasing age.

The inflection point for the risk probability of coronary stenosis ≥50% was observed within a CACS range of 63.3–66.0. The risk probability exhibited a significant increase after the inflection point, reaching its peak at 230.8, and subsequently stabilized. When the CACS ranged from 66 to 230.8, there was a progressive increase in the risk probability of coronary stenosis ≥50%. The risk probability of developing coronary stenosis ≥50% remained consistent as the CACS increased beyond 230.8. Previous studies have indicated a positive correlation between higher CACS and an increased likelihood of coronary stenosis [24–26]. This study further suggested that a CACS of 63.6–66 is an inflection point for coronary stenosis ≥50%. Clinicians should be cautious of coronary stenosis when CACS exceeds this threshold. CACS <63.6 serves as a protective factor against coronary stenosis ≥50%, and further investigation is required to understand its mechanism.

The assessment of coronary stenosis may exhibit some differences between CAG and CCTA. This variation depends on factors such as the patient population, operator experience, and technological nuances [27, 28]. We need to carefully consider these factors for a more accurate evaluation of coronary artery health.

Limitations of the analysis include the following: (1) This retrospective study included patients who had indications for CAG, potentially introducing selection bias. (2) The sample size in this study was limited. (3) This study did not further investigate the relationship between vascular-based CACS, the degree of stenosis, and the nature of plaques. Indeed, the total CACS does not offer a distinct evaluation of calcification in individual coronary vessels and is unable to detect noncalcified plaques.

## 4. Conclusions

The utilization of CACS is beneficial in detecting coronary artery stenosis. High CACS cutoff values exhibit high specificity and PPV in the diagnosis of coronary stenosis ≥50% and ≥70%. The presence of CACS = 0 does not consistently exclude the possibility of coronary stenosis ≥50% or ≥70%, especially in young patients. The inflection point for risk probability of coronary stenosis ≥50% shifts from being a protective factor to a risk factor is observed within the CACS range of 63.3 to 66.0.

## Figures and Tables

**Table 1:** Clinical characteristics of the study population.

Clinical characteristics	Total
Age, years	65 (58–71)
Men	138 (66.3%)
Systolic blood pressure (mmHg)	134.55 ± 22.92
Diastolic blood pressure (mmHg)	76.27 ± 13.87
Heart rate (times/min)	76 ± 16
BMI	24.22 (21.78–26.58)
History of smoking	84 (40.4%)
Diabetes mellitus	50 (24.0%)
Hypertension	130 (62.5%)
Positive family history	0
Hb (g/L)	134.75 ± 16.31
NTProBNP (pg/ml)	498 (174–1554)
eGFR	79.29 (64.62–91.01)
Creatinine (*μ*mol/L)	84.0 (69.70–99.90)
Total cholesterol (mmol/L)	4.82 ± 1.40
HDL (mmol/L)	1.07 (0.89–1.26)
LDL (mmol/L)	3.00 (2.24–3.59)
TG (mmol/L)	1.54 (1.02–2.36)
TnI (ng/ml)	0 (0–0.02)
Calcium (mmol/L)	2.27 ± 0.11
EF (%)	69 (62–75)
CACS	
0	32 (15.4%)
1–30	35 (16.8%)
31–100	32 (15.4%)
101–400	62 (29.8%)
401–800	25 (12.0%)
801–1300	13 (6.3%)
>1300	9 (4.3%)
Coronary artery disease	
No significant stenosis	34 (16.3%)
1-vessel stenosis ≥50%	80 (38.5%)
2-vessel stenosis ≥50%	49 (23.6%)
3-vessel stenosis ≥50%	33 (15.9%)
4-vessel stenosis ≥50%	4 (1.9%)
1-vessel stenosis ≥70%	83 (39.9%)
2-vessel stenosis ≥70%	29 (13.9%)
3-vessel stenosis ≥70%	2 (1.0%)
4-vessel stenosis ≥70%	0

Values are mean ± SD, n (%), or median (interquartile range). BMI, body mass index; Hb, hemoglobin; NTproBNP, N-terminal pro-B-type natriuretic peptide; eGFR, estimated glomerular 5ltration rate; HDL, high-density lipoprotein cholesterol; LDL, low-density lipoprotein cholesterol; TG, triglyceride; TnI, cardiac troponin-I; EF, ejection fraction; CACS, coronary artery calcium score.

**Table 2:** Different cutoff values of a calcium score to detect coronary stenosis ≥50% and ≥70%.

CACS cutoff value	Percentage of stenosis	Number (%) of patients	Sensitivity (%)	Specificity (%)	PPV (%)	NPV (%)	Accuracy (%)
0	≥50	16 (7.6)	9.6	61.9	50	14.8	20.2
	≥70	10 (4.8)	7.9	73.2	31.3	34.1	33.7

≥1	≥50	147 (70.0)	89.2	40.5	85.5	486	79.3
	≥70	114 (54.3)	90.5	28	65.9	65.7	65.9

≥30	≥50	122 (58.1)	74.1	57.1	87.2	35.8	70.7
	≥70	95 (45.2)	75.4	43.9	67.4	53.7	63

≥50	≥50	111 (52.9)	67.5	69	89.6	34.9	67.8
	≥70	86 (41.0)	68.3	52.4	68.8	51.8	62

≥100	≥50	98 (46.7)	59.6	76.2	90.8	32.3	63
	≥70	77 (36.6)	61.1	61	70.6	50.5	61.1

≥200	≥50	75 (35.7)	45.8	85.7	82.7	28.6	53.8
	≥70	61 (29.0)	48.4	74.4	74.4	48.4	58.7

≥300	≥50	56 (26.7)	34.3	95.2	96.6	26.8	46.6
	≥70	47 (22.4)	37.3	85.4	79.7	47	56.3

≥400	≥50	44 (21.0)	27.1	95.2	95.7	24.8	40.9
	≥70	38 (18.1)	30.2	89	80.9	45.3	53.4

≥1000	≥50	13 (6.2)	7.8	97.6	92.9	21.1	26
	≥70	10 (4.8)	7.9	95.1	71.4	40.2	42.3

≥1300	≥50%	9 (4.3)	5.4	100	100	21.1	24.5
	≥70%	7 (3.3)	5.6	97.6	77.8	40.2	41.8

≥1800	≥50%	2 (0.10)	1.2	100	100	20.4	21.2
	≥70%	2 (0.10)	1.6	100	100	39.8	40.9

## Data Availability

The data that support the findings of this study are available from the corresponding author upon reasonable request.
